# Dietary phytochemical index and overweight/obesity in children: a cross-sectional study

**DOI:** 10.1186/s13104-020-04979-6

**Published:** 2020-03-05

**Authors:** Omid Eslami, Mahdi Khoshgoo, Farzad Shidfar

**Affiliations:** Department of Nutrition, School of Public Health, Iran University of Medical Sciences, Tehran, Iran

**Keywords:** Dietary phytochemicals, Antioxidants, Pediatric obesity, Overweight, Body mass index

## Abstract

**Objective:**

The aim of the present study was to examine the relationship between the dietary phytochemical index (DPI) and overweight/obesity in children. This cross-sectional study was comprised of 356 children aged 7 to 10 years-old study in the city of Tehran, Iran. The dietary intake of participants was collected using a validated food frequency questionnaire. The DPI was calculated based on the daily energy derived from phytochemical-rich foods. The definition of overweight and obesity was based on the criteria developed by the US Chronic Disease Center for prevention and health promotion.

**Results:**

The overall prevalence of overweight/obesity was 35.1%. The mean (standard deviation) of the DPI was 14.25 (4.13), 24.12 (2.64), 35.41 (3.62) and 61.52 (16.47) in the first, second, third and fourth quartiles (Q), respectively. Subjects in the higher quartiles of DPI had a significantly higher intake of dietary fiber, vitamin C, and potassium compared to those in the lower quartiles. In the multiple regression analysis, subjects in the highest quartile of DPI had significantly lower odds of being overweight/obese compared to those in the first quartile [odds ratio and 95% confidence intervals for Q4 vs. Q1: 0.47 (0.25, 0.87); *P* for trend = 0.02].

## Introduction

The global prevalence of overweight and obesity in children and adolescents has significantly increased over the last four decades, exceeding 340 million in 2016 [1]. Childhood obesity has emerged as serious public health concern since it has been linked to several adverse health outcomes in the later ages including metabolic syndrome, type 2 diabetes, coronary artery diseases, non-alcoholic fatty liver disease, and certain cancers [2–6]. It is a multifactorial phenomenon caused by a complex interaction between genetic, lifestyle and environmental factors, which of these, diet, as a modifiable component of lifestyle, has been shown to play a critical role [7]. Accumulating evidence indicates that the diet quality of children and adolescents has undergone an unfavorable shift toward the consumption of high energy-dense foods, while low intake of nutrient-dense sources including fruits and vegetables [8, 9]. Such low-quality diets have been linked to a higher risk of childhood obesity [10]. Therefore, dietary modifications are recommended as the main approach to the prevention and treatment of childhood obesity [11].

In recent years, dietary anti-oxidants, particularly phytochemicals have gained a lot of attention in regard to their effect on adiposity. Phytochemicals are referred to as non-nutritive bioactive compounds including polyphenols (phenolic acids, flavonoids, isoflavones, lignans, stillbenes, curcuminoids, and calcones), Terpenoids, Organosulfurs, and phytosterols. These compounds are highly found in plant-based foods particularly fruits and vegetables [12]. The association between dietary intakes of certain phytochemicals’ subtypes with adiposity was already investigated in some epidemiological studies. For example, an inverse association has been reported between the habitual intake of flavonoids and risk of overweight/obesity or changes in adiposity measures [13–15]. The exact mechanisms underlying the possible beneficial effect of phytochemicals on body weight are not fully understood; however, evidence from invitro and invivo studies proposed that phytochemicals might reduce adiposity and body fat by several mechanisms including increasing lipolysis, reducing lipogenesis and proliferation as well as decreasing proinflammatory mediators in adipocytes, improving insulin sensitivity, inducing apoptosis, suppressing lipogenesis and angiogenesis [16]. Therefore, it is hypothesized that a diet rich in phytochemicals could reduce the risk of excess body weight.

Nevertheless, most of the studies have focused on a particular type of phytochemicals such as flavonoids, not the whole content of phytochemicals in the diet. Regarding the fact that quantification of phytochemicals in food sources is expensive and impractical for large epidemiological studies, using the dietary phytochemical index (DPI), as an alternative method, has been proposed by McCarty [17]. This index, which is defined as the percentage of energy intake derived from phytochemical-rich foods, provides a simple and practical way to estimate the quality of diets in clinical practice as well as to optimize eating pattern by monitoring the intake of foods rich in phytochemicals during a dietary intervention program [17]. To date, all studies that have used the DPI were conducted in the adult population and there is limited evidence in children and adolescents. With these regards, the present study was conducted to assess the association of the dietary load of phytochemicals using DPI with the risk of overweight and obesity in children.

## Main text

### Methods

The present cross-sectional study was comprised of children aged 7 to 10 years attending the elementary schools in the city of Tehran, Iran, in 2018. The participants were chosen using the stratified cluster sampling method; the city of Tehran was divided into five geographic regions including the north, south, east, west, and center. Of the list of all primary schools in each district, four public schools were randomly chosen. Thereafter, within each school, a total of 18 to 20 students were randomly selected. The children’s parents were invited to participate in the briefing session in which the objectives of the study were explained to them, and those who wish to participate in the study signed written informed consent. All participants were ensured that their data would remain confidential. The Ethics Committee of Iran University of Medical Sciences confirmed the study protocol (code no. IR.IUMS.REC1395.9413323002). All children aged 7 to 10 years old who had not a history of chronic diseases including cardiovascular, pulmonary, gastrointestinal, and renal diseases, as well as autoimmune diseases, diabetes, and cancer as well as those who had not followed any special diets during the past year such as gluten-free, lactose-free, or weight-loss diets, were eligible to participate in this study.

Anthropometric measurements were performed by a trained dietitian using calibrated equipment. Weight was measured using Seca scale with an accuracy of 100 g with light clothes and without shoes. Measurement of height was done using stadiometer with an accuracy of 0.5 cm without shoes. Body mass index (BMI) was calculated as weight (kg) divided by square of height (m^2^). The diagnosis of overweight and obesity was based on the criteria developed by US Chronic Disease Center (CDC) for prevention and health promotion; Subjects with BMI percentile ≥ 85 and < 95 were considered overweight, and those with BMI ≥ 95 were obese [18]. Physical activity was assessed using a validated 19-item questionnaire, which investigates the frequency, severity, and duration of several kinds of activities that are usually conducted by children inside and outside of the school.

A validated 168-item food frequency questionnaire (FFQ) was used for dietary assessment [19]. For each item, a trained dietitian asked the children’s parents to report the portion size and frequency of food consumption on a daily, weekly, and monthly basis in the previous year. Then, the frequency of food intakes was converted to the daily and also the reported portion sizes were converted to grams using household measures. The energy and nutrient intake were determined using Nutritionist IV software. The method developed by McCarty was used for calculation of DPI, which was as follows: DPI = (daily energy derived from phytochemical-rich foods (kcal)/total daily energy intake (kcal)) × 100 [17]. Phytochemical-rich foods were considered whole grains, fruits, vegetables, soy products, legumes, nuts, seeds, as well as olive oil, tomato sauces, and pure fruit and vegetable juices. Among the plant-based food, potatoes were not included due to their low content of phytochemicals.

Data analysis was performed using SPSS software version 22 (IBM Corp., Armonk, NY, USA). To assess the differences in the continuous variables across the quartiles of DPI, the ANOVA test was used, while categorical variables were compared using the Chi Square test. Also, a logistic regression model was applied to determine the odds ratio (OR) and 95% confidence intervals (%95 CI) for overweight and obesity across the DPI quartiles. A *P* value < 0.05 was considered as statistically significant.

### Results

A total of 356 eligible children with a mean (standard deviation [SD]) age of 8.61 (1.01) were included in this study (53.6% boys). The prevalence of overweight and obesity was 21.6% and 13.5%, respectively. The mean (SD) of the DPI was 14.25 (4.13), 24.12 (2.64), 35.41 (3.62) and 61.52 (16.47) in the first, second, third and fourth quartiles, respectively. As shown in Table 1, the distribution of participants in terms of age, sex, weight, height, and physical activity score were not significantly different across the quartiles of DPI.

**Table 1:** General characteristics of study participants across the quartiles (Q) of the dietary phytochemical index (DPI)

Variables	DPI quartiles					*P*-value
	Total (N = 356)	Q1 (N = 89)	Q2 (N = 89)	Q3 (N = 89)	Q4 (N = 89)	
Age (years)						
7–8	170 (47.8)^a^	37 (21.8)	37 (21.8)	48 (28.2)	48 (28.2)	0.14^b^
9–10	186 (52.2)	52 (28)	52 (28)	41 (22)	41 (22)	
Sex						
Male	190 (53.4)	45 (23.7)	52 (27.4)	44 (23.2)	49 (25.8)	0.60^b^
Female	166 (46.6)	44 (26.5)	37 (22.3)	45 (27.1)	40 (24.1)	
Weight (kg)	28.89 ± 5.76^c^	29.72 ± 6.46	29.11 ± 5.49	28.09 ± 5.40	28.63 ± 5.58	0.27^d^
Height (cm)	127.75 ± 8.25	127.79 ± 9.02	128.96 ± 7.71	126.96 ± 8.26	127.28 ± 7.95	0.39^d^
Physical activity (score)	3.10 ± 0.38	3.10 ± 0.38	3.09 ± 0.43	3.10 ± 0.36	3.11 ± 0.35	0.99^d^

^a^Data are presented as frequency (percentage)

^b^Obtained from the Chi square test

^c^Data are presented as mean ± standard deviation

^d^Obtained from ANOVA

Table 2 compares the intake of energy and nutrients across the quartiles of DPI. Subjects in the higher quartiles of DPI had a significantly higher intake of dietary fiber (*P *= 0.003), vitamin C (*P *< 0.001), and potassium (*P *< 0.001) compared to those in the lower quartiles. While, intake of energy, as well as carbohydrate, protein, total fat, folate, calcium, and magnesium, were not significantly different between the DPI quartiles.

**Table 2:** Dietary intakes of study participants across the quartiles of DPI

Variable	DPI quartiles					*P*-value^*^
	Total (N = 356)	Q1 (N = 89)	Q2 (N = 89)	Q3 (N = 89)	Q4 (N = 89)	
Energy (kcal/d)	2244.97 ± 41.66^a^	2226.30 ± 82.96	2318.63 ± 77.99	2278.28 ± 83.31	2156.70 ± 89.06	0.55
Carbohydrate (g/d)	324.71 ± 6.85	297.55 ± 12.73	335.24 ± 11.07	337.03 ± 14.72	329.04 ± 15.68	0.14
Protein (g/d)	73.95 ± 2.38	76.62 ± 6.32	71.67 ± 2.79	74.23 ± 4.12	73.27 ± 5.21	0.90
Total fat (g/d)	88.09 ± 3.42	99.04 ± 10.83	87.71 ± 4.13	84.91 ± 5.29	80.69 ± 4.98	0.26
Fiber (g/d)	19.96 ± 0.63	16.54 ± 1.45	18.98 ± 0.72	22.18 ± 1.33	22.15 ± 1.38	*0.003*
Folate (mg/d)	327.69 ± 10.45	293.39 ± 18.12	344.87 ± 18.30	343.38 ± 24.69	329.12 ± 21.72	0.27
Vitamin C (mg/d)	174.35 ± 6.76	111.61 ± 6.16	166.14 ± 9.32	205.81 ± 14.18	213.86 ± 18.39	< *0.001*
Calcium (mg/d)	1036.87 ± 26.17	939.72 ± 39.15	1077.89 ± 53.41	1101.32 ± 51.51	1028.55 ± 61.97	0.13
Magnesium (mg/d)	287.94 ± 7.39	264.77 ± 17.14	284.23 ± 10.03	303.61 ± 15.90	299.17 ± 14.94	0.23
Potassium (mg/d)	3504.36 ± 85.42	2849.18 ± 118.39	3461.85 ± 137.35	3731.66 ± 169.67	3974.76 ± 219.24	< *0.001*

Italic values indicate the significance of *P*-values (*P*-value < 0.05)

^a^Data are presented as mean ± standard deviation

^*^ Obtained from ANOVA

Crude and multivariable-adjusted ORs with 95% CI for overweight and obesity in the whole study population across the quartiles of DPI are presented in Table 3. In the crude model, subjects in the highest quartile of DPI had significantly lower odds of being overweight/obese compared to those in the first quartile (OR and 95% CI for Q4 vs. Q1: 0.49 (0.26, 0.90); *P* for trend = 0.03). Moreover, this association remained significant in the fully adjusted model controlling for potential confounders including age, sex, energy intake and physical activity (OR and 95% CI for Q4 vs. Q1: 0.47 (0.25, 0.87); *P* for trend = 0.02).

**Table 3:** Crude and adjusted odds ratio (95% confidence intervals) for overweight/obesity across quartile of DPI

Overweight and obesity	DPI quartiles										*P*-trend*
	Q1 (N = 89)	Q2 (N = 89)			Q3 (N = 89)			Q4 (N = 89)			
		OR	95% CIs		OR	95% CIs		OR	95% CIs		
			Lower	Upper		Lower	Upper		Lower	Upper	
Crude	1	0.40	0.21	0.74	0.44	0.24	0.82	0.49	0.26	0.90	*0.03*
Adjusted^a^	1	0.39	0.21	0.74	0.43	0.23	0.81	0.47	0.25	0.87	*0.02*

Italic values indicate the significance of *P*-values (*P*-value < 0.05)

*OR* odds ratio, *CIs* confidence intervals

^a^ Adjusted for age, sex, energy intake and physical activity

^*^Obtained from the logistic regression analysis

### Discussion

The present study found that a higher load of phytochemicals in the diet was inversely associated with the risk of being overweight/obesity in children. Higher DPI was also accompanied by a better diet quality characterized by a higher intake of dietary fiber, vitamin C and potassium in the study population.

Previous studies have reported an inverse link between dietary DPI and risk of chronic diseases such as insulin resistance [20], prediabetes [21], depressive symptoms, anxiety, and psychological distress [22], breast cancer [23], hypertension [24], hypertriglyceridemia [25]. To date, a limited number of studies have assessed the association between dietary DPI and obesity; however, all have been conducted in the adult population. In a cross-sectional study by Vincent et al. [26] among 54 US adults aged 18 to 30 years, a significant inverse correlation was found between DPI score with BMI, waist circumference (WC), waist-to-hip ratio, and body fat percent.

Similarly, two studies [25, 27] conducted among a sample of Tehranian adults aged 19 to 70 years-old recruited from the Tehran Lipid and Glucose Study have reported consistent results in regards to the association between DPI and obesity. Bahadoran et al. [25] showed that in a cross-sectional analysis among 2567 subjects, in the higher quartiles of DPI had 66% lower odds of abdominal obesity, determined by high WC, compared to those in the lower quartile. Furthermore, Mirmiran et al. [27] in a longitudinal analysis among 1938 adults recruited from the same population, reported a negative association between DPI with 3-year changes in weight. The finding of our study also is in line with the above-mentioned ones. However, the current evidence is too limited to draw a definite conclusion on this topic. Moreover, due to the great heterogeneity between studies in terms of sample size, design, the type of outcome, the age and socio-demographic characteristics of study participants, made it difficult to generalize these findings to the other population.

To the best of our knowledge, this is the first study, in its kind, that has been conducted among children. Instead to rely on a particular type of phytochemicals, the present study reported the load of total phytochemicals in the diet, which could better reflect the health effects of these compounds. Also, another strength of this study was using a validated FFQ, which enables to assess the dietary intakes during a long-term period.

### Conclusion

In summary, a diet loaded with high phytochemical-rich foods was associated with a lower risk of overweight/obesity in a sample of school-aged children in Iran. Further studies particularly the prospective ones are needed to confirm these findings.

## Limitations

First, because of the cross-sectional design of this study, it was not possible to determine the direction of the relationship. Second, data on dietary intakes were investigated by the self-reporting method, and the fact the results might be affected by the recall bias could not be neglected. Third, this study was comprised of a sample of school-aged children within a limited age range in the capital of Iran. Whether the findings of the study are generalizable to children living in other geographical regions with different dietary habits remained to be answered. Finally, the DPI used in this study had some weaknesses; it did not consider the type of phytochemicals consumed and just quantify the load of dietary phytochemicals using the energy content of their food sources. Furthermore, some foods such as green and black tea are a rich source of phytochemicals, but they are not considered in DPI since they have no contribution to the energy intake.

## Data Availability

The datasets used during the current study are available from the corresponding author on reasonable request.

## Abbreviations

DPI: Dietary phytochemical index
BMI: Body mass index
CDC: US Chronic Disease Center
FFQ: Food frequency questionnaire
OR: Odds ratio
CI: Confidence interval
SD: Standard deviation
WC: Waist circumference
